# Embracing smart wellness: Exploring perceptions of preventive healthcare technology in the digital era

**DOI:** 10.12688/f1000research.160126.1

**Published:** 2025-01-24

**Authors:** Debanjali Jairam, Swathi K S, Smitha Nayak

**Affiliations:** 1 Prasanna School of Public Health, Manipal Academy of Higher Education, Manipal, Karnataka, India; 2 Manipal Institute of Technology, Manipal Academy of Higher Education, Manipal, Karnataka, India

**Keywords:** smart healthcare, preventive health, digital health, technology adoption

## Abstract

**Background:**

Technology is revolutionizing healthcare, making it more connected, efficient, and patient-centric. Smart healthcare tools like wearables and mobile health applications empower individuals to manage their health proactively. By leveraging these technologies, individuals can monitor chronic conditions like hypertension, diabetes, obesity, and cardiac issues, potentially preventing their detrimental consequences. This proactive approach not only enhances personal health management but also contributes to the overall well-being of society.

**Objective:**

This study aims to understand individuals’ perceptions of smart technology usage and identify the antecedents influencing the adoption of smart healthcare technological applications.

**Methods:**

This cross-sectional study used a structured questionnaire to collect the responses from 390 respondents in the Indian context. The data were analyzed using Partial Least Square Structural Equation Modeling.

**Findings and Conclusion:**

The findings of this research show that antecedents such as self-efficacy, preventive awareness, technology promptness and innovativeness, and social influence play a significant role in the adoption of technology among individuals. Further, the study’s results will help to develop and promote technological applications to improve population health and have implications for healthcare providers, technology developers, marketers, and researchers.

## 1. Introduction

Health and health care are considered the most important elements of life. Inevitably, the current healthcare systems are experiencing extensive stress due to the continually increasing longevity of the population and the associated growth in chronic illnesses ( Andersen, 2023; Sharma & Popli, 2023). Along with other service sectors, healthcare has also experienced a profound change in digitalization and artificial intelligence due to emerging medical advancement and customer demand for exceptional standards of healthcare service ( Alowais et al., 2023). With the changing healthcare approach, healthcare focuses on transforming from reactive healthcare to more proactive and personalized ( Chawla, 2020). The usage of Smart Healthcare Technologies is widely accepted across the globe. Moreover, wearable gadgets, healthcare apps, and teleconsultation have become essential for daily usage. It enables both patients and clinicians to be constantly vigilant about their health risk of sickness and to take specific preventative steps depending on the results of their monitoring ( Massaro, 2023; Tian et al., 2019). Smart healthcare aims to assist patients by informing them about their health and medical conditions ( Rani et al., 2023). Smart healthcare makes it simpler to utilize the resources that are currently accessible. It lowers the user’s healthcare costs and aids patient remote monitoring. Additionally, it enables medical professionals to offer their services anywhere in the world ( Chau et al., 2019; Sundaravadivel et al., 2017). Smart Healthcare includes diverse innovation while using mere technological advances. With the advancement of medical digital technologies, it focuses more on patients rather than the traditional disease-centric protocols. The term “smart healthcare” describes a health service system that proactively monitors and intelligently responds to the needs of the healthcare environment. Smart healthcare connects people, resources, and healthcare organizations using wearables, IoT, and mobile Internet technologies. Smart healthcare can improve decision-making, ensure participants receive the needed services, promote communication between all healthcare stakeholders, and ensure utilization. optimize resources. The delivery of smart healthcare services involves many individuals, including doctors, patients, hospitals, and research organizations ( Tian et al., 2019; Kraus et al., 2021).

Many diverse tactics and efforts are being used by health professionals and academics from many disciplines, including healthcare management science, health policy, and innovation management, to modernize healthcare delivery and prevent chronic health conditions. “Preventive medicine is the practice of promoting preventive health care to improve patient well-being.” The primary objective is to prevent illnesses, impairments, and fatalities ( American College of Preventive Medicine, 2019). According to the Ottawa Charter, “the process that enables people to increase control over their health and improve their overall health” is known as “health promotion” ( World Health Organization [WHO], 2020). Initiatives promoting health prioritize overall well-being and aim to forestall illnesses rather than predominantly concentrating on individuals with a heightened risk of specific diseases ( Sibeudu, 2022). These diseases require regular monitoring to intervene if any parameters are elevated promptly. Daily lifestyle changes can help improve health conditions and halt the onset of these diseases. Early detection and intervention would help curb the parameters ( Merck, 2017). Studies on the compulsion to use smart health technology by patients with chronic diseases owing to the extremity of their ailments have been done in the past. On the contrary, less is known about thriving individuals and their desire to sustain that state while attempting to prevent future ailments ( Bettiga et al., 2020). Presently, people already engage in self-care. Wearables and mobile health applications are examples of self-monitoring smart healthcare technologies ( Kreitmair, 2023; Stoumpos et al., 2023). Wearable technology can be crucial for tracking the physiological data of people with disabilities or long-term conditions **(** Partheniadis & Stavrakis, 2019; Koo & Fallon, 2018), as well as for lowering mortality and hospitalization rates in less developed nations where chronic diseases are more common ( Binyamin & Hoque, 2020). Due to regional differences in consumer cultural traits, technological adoption preferences, or both ( Chiu & Cho, 2021; Meier et al., 2020 **)**, consumer behaviour connected to wearable technology may vary.

As emphasized earlier, supporting healthcare efforts and prioritising disease prevention are extremely important in today’s culture. In order to better understand the factors impacting the general public’s behavioural propensity to embrace smart healthcare technology for illness prevention and healthcare promotion, the study set out to investigate the Indian perspective. The main aim was to find out how people felt about using smart technology, identify the variables influencing people’s intentions to use smart health technology, and identify the elements driving the adoption of smart healthcare applications.

Among other smart healthcare technologies, despite many contributing factors of mobile health, some challenges impeded their successive usage, including legislative problems, stakeholder disputes, and technological constraints. These obstacles imply that providers of services are still uncertain about how to deliver mobile health to the market **(** PwC, 2013). Due to the intelligence and intercommunication capabilities of its qualities, convenience is one of the significant relative benefits of adopting mobile health technology in comparison to conventional medical and healthcare services ( Gao et al., 2015; Iyanna et al., 2022; Yoon & Kim, 2008 **)**. Guner and Acarturk (2020) suggested that the utilization of information and communication technology (ICT) by elderly individuals holds the potential to enrich their quality of life. Hence, this study enables us to better understand the preferences and expectations, enabling developers to enhance user experience and engagement with smart technologies. It also helps us to identify psychological, social, and technical barriers that impede the widespread adoption of smart health technologies.

The research paper is structured as follows: An explanation of the literature review and hypothesis development is presented first, followed by the methodology adopted for this study, which is explained in detail. The subsequent section explains the findings, practical implications, and suggestions for future studies.

## 2. Review of literature and hypotheses development

The theoretical underpinnings are borrowed from the Technology Acceptance Model (TAM) ( Davis, 1989). The Technology Acceptance Model (TAM) accounts for the unique use of smart healthcare technology for preventative healthcare ( Holden & Karsh, 2010). The two crucial elements of the “TAM” model are, **“perceived usefulness (PU)”** and **“perceived ease of use (PEOU),”** impacted by external influences. Both factors influence “Behavioural Intention to use (BI)” and “Attitude Towards using (ATT),” with BI being influenced by ATT. According to Davis (1989), perceived usefulness is “the degree to which a person believes that using a particular system would enhance his or her job performance”, and perceived usability is “the degree to which a person believes that using a particular system would be free of effort.”

TAM essentially holds that individual acceptance occurs in three stages: (i) external factors that relate to people’s beliefs influence their perceptions of the usefulness and usability of an IT system; (ii) the perceived usefulness and usability of the system influences behaviour; and (iii) the behaviour influences the actual use of the IT system. The pillars of the theoretical framework are PU and PEOU.

Behavioural Intention is the central decision-making construct of the model used in this study. The conceptual framework of this study represents Prevention Awareness, Self-Efficacy, Technology Promptness, and Innovativeness as the antecedents. Prevention awareness and self-efficacy are the antecedents of the determinant of perceived usefulness, and technological promptness and innovativeness are the antecedents of perceived ease of use.

Subjective Norms have been included as one of the main determinants of the model. The subjective norm reflects the social pressure that results from trying to live up to others’ expectations. Being a part of society, people may get advice and pressure from friends, family, coworkers, and peers regarding their lifestyle and health choices. Understanding how these external factors affect the adoption of mobile health advances is significant ( Bettiga et al., 2020).

**Perceived Usefulness** is the extent to which a person thinks employing a particular system would improve his or her performance. According to ideas regarding consumption values, consumers’ choices are mostly influenced by functional values ( Davis, 1989). It denotes the value obtained from successfully completing a task and is connected to superiority over alternatives. Studies on the adoption of new technologies have lately presumed the relationship between perceived usefulness and intention to adopt since it has been carefully studied and verified by prior research with various settings, technologies, and consumer groups ( Ahmad, 2014; Edmunds et al., 2012; Indu & Raj, 2012). Consequently, the following hypothesis has been formulated:

*H* _*1*_ *:*
***Perceived usefulness has a positive impact on behavioural intention to adopt smart technology.***

Perceived ease of use is the idea that using a particular technology would be effortless. It is an inducer of personal technology usage. PEOU has a critical role in influencing the adoption of innovations since it may evaluate various technologies in terms of their time savings ( Collier & Kimes, 2013). PEOU has received widespread confirmation of its significance in forecasting technology adoption and is seen as a valuable indicator of a technology’s functional properties ( Davis & Venkatesh, 2004).

*H* _*2*_ *:*
***Perceived ease of use has a positive impact on behavioural intention to adopt smart technology.***

The term “subjective norm” refers to an individual’s assessment of the extent to which significant others endorse or disapprove of the target activity. Individuals do participate in intricate social networks and communities, which may impact their intents and behaviours ( Fishbein et al., 2007). Social influence can originate from numerous avenues, including fellow consumers of the same business or similar services, as shown in feedback, evaluations, and positive experiences. People engage in complex social networks and groups, which may influence their intentions and actions ( Bettiga et al., 2020). Thus, the following hypothesis has been formulated:

*H* _*3*_ *:*
***Subjective norms has a positive impact on behavioural intention to adopt smart technology.***

The healthcare systems of the most developed nations focused more on prevention due to the drop in chronic patients and hospitalizations. Further, TAM2 ( Venkatesh & Davis, 2000) reveals the influence of subjective norms and social influence on intentions to use. In developing countries, though, people are aware of disease prevention, and that drive must endure for years or even decades to experience the benefit of prevention ( Champion & Skinner, 2008; Mosca et al., 2010). Making people aware of the consequences of diseases and how using smart healthcare can be beneficial in preventing such health issues could influence the adoption of the same. So, the subsequent hypothesis has been developed:

*H* _*4*_ *:*
***Prevention awareness has a positive impact on perceived usefulness.***

Self-efficacy is the conviction that one can independently do tasks or get desired results. Zhang et al. (2017) found that perceived usefulness has a more significant impact on adoption intention when a user has a high degree of self-efficacy when utilizing m-Health services. The influence of perceived utility on the intention to adopt is highlighted when users possess a strong self-efficacy in using novel technology services. On the other hand, a user who is unconfident in their capacity to utilize new technology efficiently might feel demoralized and choose not to adopt it ( Zhang et al., 2017).

*H* _*5*_ *:*
***Self-efficacy has a positive impact on perceived usefulness.***

Technology promptness is believed to be a relevant antecedent of perceived ease of use because it makes access and usage easier. The concept of perceived technical promptness conveys all the underlying conditions that underpin technology usage, such as its prompt availability when required or other contextual drivers of use. Technology makes it easier for consumers to access and utilize services quickly in terms of time and geography ( Mallat et al., 2009).

*H* _*6*_ *:*
***Technology promptness has a positive impact on perceived ease of use.***

Innovativeness is a psychological condition shaped by cognitive facilitators that impact a person’s inclination to integrate new technologies. Perceived ease of use might be influenced by an individual’s ideas about cutting-edge technology when assessing the qualities of newly developed products or services and contemplating adopting them ( Lin et al., 2007; Schwarz & Ernst, 2009).

*H* _*7*_ *:*
***Innovativeness has a positive influence on perceived ease of use.***

Forecasting a person’s interest in utilizing the system in the future is possible by looking at their intention, which is their choice or plan to carry out a specific act independently. By assessing the constructs that are the antecedents and determinants of the model, the behavioural intention of people and their decision-making to adopt smart healthcare technologies could be identified.

**Figure 1.**
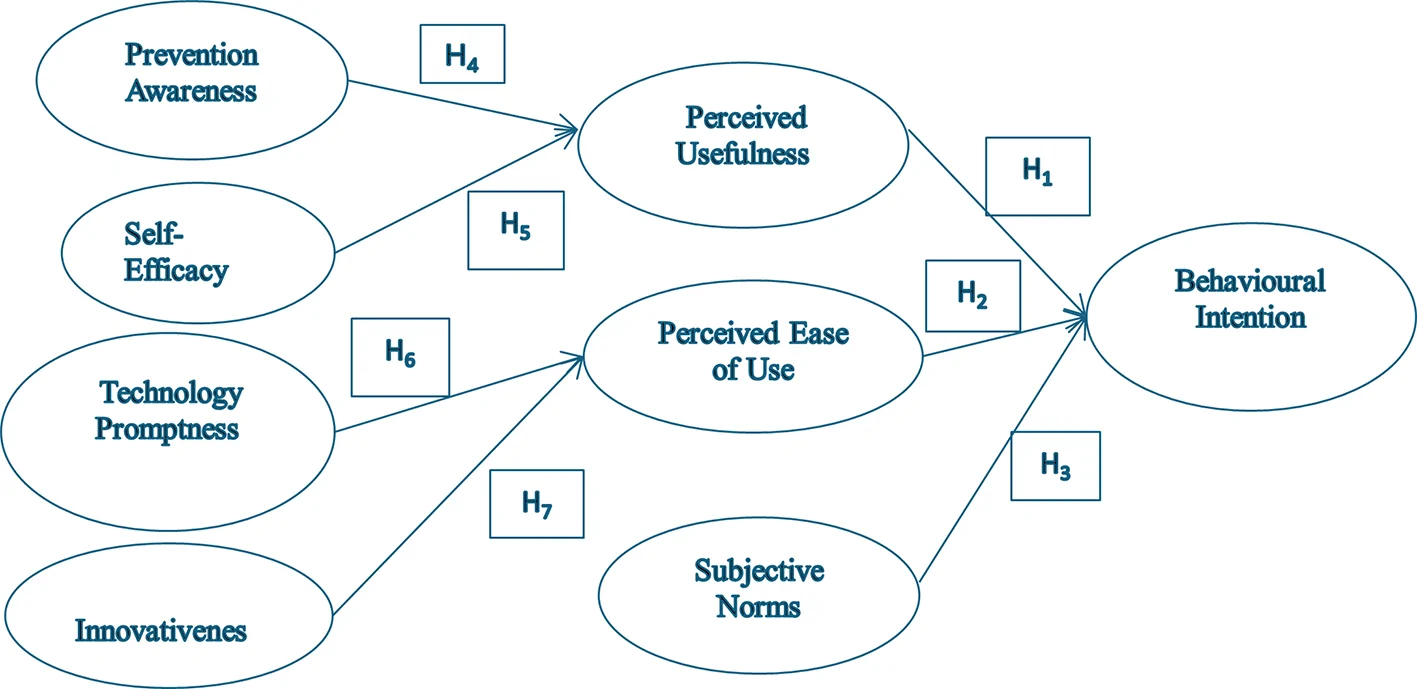
Conceptual framework.

Though the researchers have studied the adoption intention in different countries worldwide, there is a dearth of literature on the Indian context. Hence, based on the literature, the researchers have proposed the following conceptual framework given in Figure 1.

## Methods

This cross-sectional empirical study aimed to assess people’s perception of adopting smart healthcare technologies like mobile health, e-health, wearables and telemedicine to prevent diseases and promote health. The study utilized a survey-based approach, where participants completed a structured questionnaire designed to assess behavioural intentions. The initial course of action was to create the survey questionnaire. The questionnaire was developed based on a review of existing literature and validated instruments where applicable. The first section of the questionnaire included questions related to the demographic particulars of the respondents, such as age, gender, and occupation. The second part of the questionnaire consisted of the constructs adapted from seminal theories, including the TAM ( Davis, 1989) and the extended Health Belief Model (HBM) ( Rosenstock et al., 1988; Ross et al., 2010). It encompassed seven independent variables: Prevention Awareness (PREV), Self-Efficacy (SE), Innovativeness (INN), Technology Promptness (TECH), Perceived Usefulness (PU), Perceived Ease of Use (PEOU), and Subjective Norms (SN). These variables were selected based on their relevance to understanding individuals’ behavioural intentions towards adopting smart healthcare technology. Specifically, the first four constructs (PREV, SE, INN, and TECH) were considered antecedents influencing individuals’ decision-making processes, while the subsequent three constructs (PU, PEOU, and SN) were identified as determinants of decision-making. The dependent variable under investigation was behavioural intention (BI), which reflects individuals’ intentions to adopt smart healthcare technologies. The questionnaire consisted of a total of 30 measurement items. A five-point Likert Scale was used to measure the items, with the scale ranging between “Strongly Disagree” being 1 and “Strongly Agree” being 5. Next, the content validity of the questionnaire was reviewed by academic experts from the healthcare and marketing management domains. Further, a pre-test of the questionnaire with a small group of participants was done to refine the language and prevent any potential confusion or ambiguous interpretations.

The study population involved the general public in India. A non-random convenience sampling method was employed, which involves collecting the data from the respondents accessible to the researcher ( Sekaran and Bougie, 2016). The inclusion criteria for respondents were people above 18 years of age who are mentally stable, can read and write English, and have a Google account. Utilizing the sample size calculation formula for an infinite population, the study aimed to recruit 385 respondents.

Following the validation of the questionnaire, the registration number for this trial was CTRI/2023/09/058164. Participants were provided with clear and comprehensive information regarding the purpose of the study. All personal data were anonymized to ensure privacy and used solely for research purposes. The researchers were able to collect a total of 398 responses. During data cleaning, eight responses were repetitive and incomplete submissions. Therefore, after data cleaning, 390 responses were considered for data analysis. The descriptive analysis of the data was done using statistical software Jamovi 2.4.11, and the model assessment and hypothesis testing were done using SmartPLS4, a multivariate data analysis technique of the second generation that enables testing of additive and linear models.

## 3. Analysis and Results

### 3.1 Demographic particulars

The study considered 390 complete responses for final data analysis. The demographic particulars of the respondents are given in Table 1. Of the total sample population, 39.2% are male, and 60.5% are female. In the age cohort distribution, 65.9 % of the respondents are 36-50 years old.

**Table 1.** Demographic details of the respondents.

Characteristics		Total No. (N)	Percentage (%)
**Gender**	Male	153	39.2
	Female	236	60.5
	Transgender	1	0.3
**Age**	18-35	104	26.7
	36-50	257	65.9
	>50	29	7.4
**Education**	10 ^th^ Pass	0	0
	12 ^th^ Pass	8	2.1
	Graduation	184	47.2
	Post Graduation and Above	198	50.8
**Occupation**	Salaried	170	43.6
	Self-Employed	25	6.4
	Professional	30	7.7
	Homemaker	19	4.9
	Retired	9	2.3
	Student	137	35.1
**Annual Household Income**	<5 Lakh	120	30.8
	5-10 Lakh	115	29.5
	11-15 Lakh	55	14.1
	>15 Lakh	100	25.6

More than 97% of the respondents were graduates and postgraduates in the education level category.

The second-generation multivariate data analysis software Smart PLS4 was used to assess the structural model and test the hypotheses ( Ringle et al., 2023). Assessing the structural model by PLS-SEM helps the researcher establish the model’s capability to predict the reliability and validity of the dependent factors or the constructs ( Hair et al., 2014).

### 3.2 Measurement model

The reliability and validity of the model have been assessed by measuring Cronbach’s alpha, composite reliability, individual indicator reliability, and average variance extracted (AVE). The findings regarding reliability and convergent validity are summarized in Table 2. All model constructs exhibited Cronbach alpha values exceeding 0.7, composite reliability values surpassing 0.8, and AVE values more excellent than the threshold of 0.5. Therefore, it ensures the reliability and validity of all the constructs of the measurement model.

**Table 2.** Construct Reliability and Validity.

Constructs	Cronbach's Alpha	Composite Reliability	AVE
**BI**	0.896	0.928	0.763
**INN**	0.730	0.844	0.645
**PEU**	0.659	0.813	0.595
**PREV**	0.861	0.915	0.782
**PU**	0.878	0.916	0.733
**SE**	0.877	0.915	0.730
**SN**	0.657	0.802	0.582
**TECH**	0.743	0.854	0.661

Further, the researchers measured the Fornell-Larcker criteria to confirm the discriminant validity of the model. Establishing discriminant validity implies that a construct is unique and captures facts not represented by other constructs in the model. It compares the square root of the AVE values with the latent variable correlations. The square root of each construct’s AVE should be greater than its highest correlation with any other construct, which ensures the discriminant validity attainment ( Hair et al., 2014). Table 3 displays the findings of the Fornell-Larcker criterion values for this research. The square root of each construct is greater than its correlation with other constructs, which indicates the presence of discriminant validity in the model.

**Table 3.** Fornell-Lacker Criterion.

	BI	INN	PEU	PREV	PU	SE	SN	TECH
**BI**	**0.874**							
**INN**	0.571	**0.803**						
**PEU**	0.562	0.575	**0.771**					
**PREV**	0.730	0.517	0.504	**0.884**				
**PU**	0.626	0.462	0.513	0.686	**0.856**			
**SE**	0.641	0.628	0.721	0.594	0.611	**0.855**		
**SN**	0.519	0.453	0.508	0.491	0.512	0.470	**0.763**	
**TECH**	0.642	0.602	0.602	0.636	0.581	0.629	0.434	**0.813**

### 3.3 Structural model assessment

**Figure 2.**
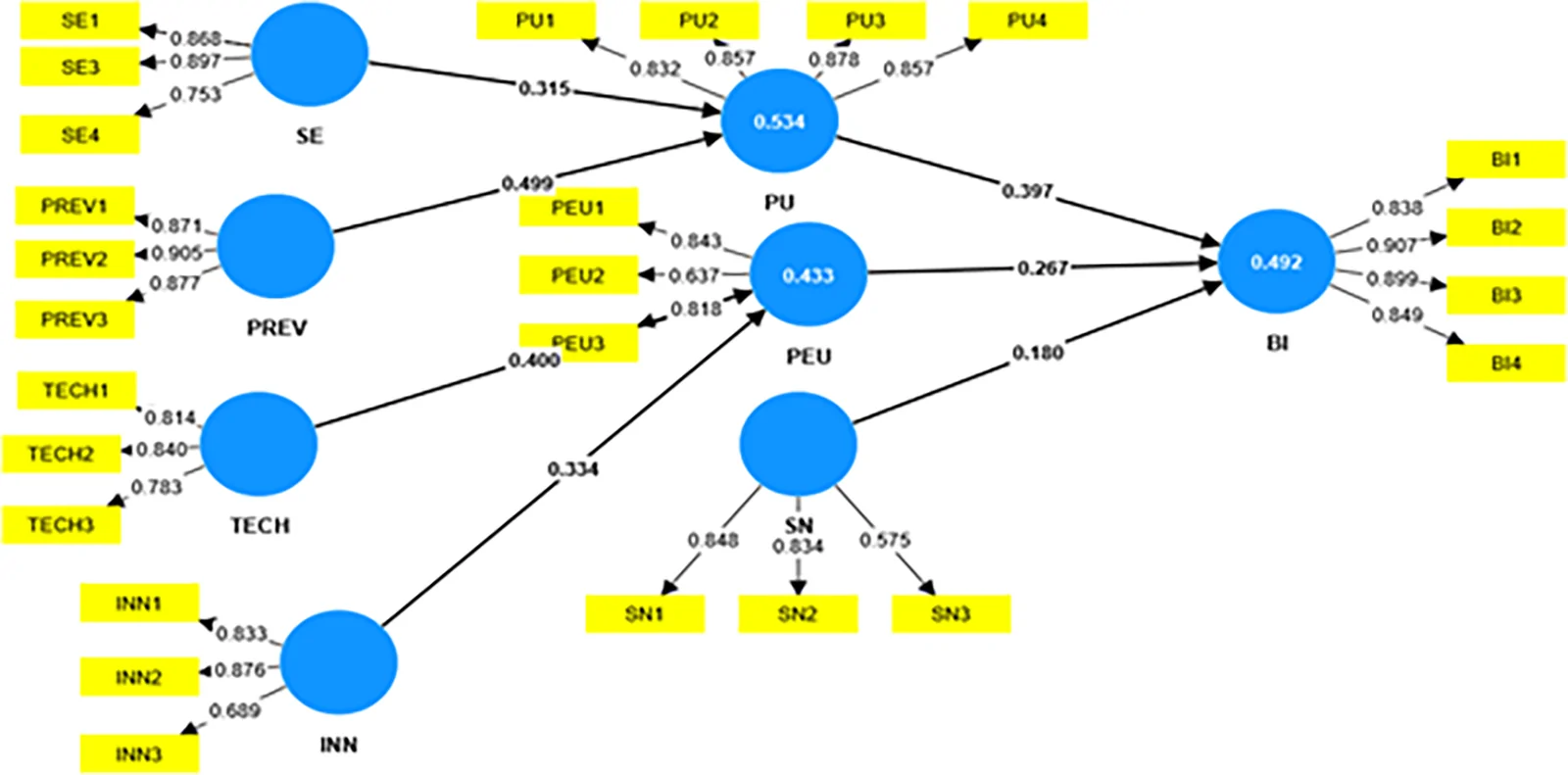
Path diagram showing the structural assessment of model.

After determining the acceptable measurement model, the researchers assessed the structural model, which involved testing the significance of path coefficients and the coefficient of determination (R ^2^ value). We performed a bootstrap analysis with 5000 samples. The estimates for the structural model relationships or path coefficients show the correlation between the constructs ( Hair et al., 2014). The coefficient of determination, or R ^2^ value, is the metric for assessing the predictive relevance of the structural model. It represents the total effect of the external latent variables on the endogenous latent variable ( Hair et al., 2014). The structural model in Figure 2 portrays the path coefficients that demonstrate the relationship between independent and dependent variables and the coefficient of determination, R ^2^ values of the endogenous constructs PE, PEU, and BI.

The path coefficient values of all the model constructs indicate stronger relationships as the values are above 0.10. The R ^2^ value of Behavioural Intention (BI) is 0.492, which explains the predictive relevance of the model. The literature states that R ^2^ values of 0.25, 0.50, and 0.75 for dependent variables denote the model’s weak, moderate, and substantial accuracy, respectively ( Becker et al., 2012; Collier & Bienstock, 2006). Here, the R ^2^ values of PU, PEU and BI are 0.534, 0.433 and 0.492, respectively, which proves the predictive relevance of the constructs in the model, confirming the research hypotheses of the study.

The t-value and p-values are used to determine the statistical significance of the parameter estimations from the structural equation modelling. In the research, the association between the independent and dependent variables is significant as the t-value is greater than the threshold value of 1.964, and the p-value is less than 0.05. The results are given in Table 4.

**Table 4.** Significance testing results of the structural model path coefficients.

Hypotheses	Relations	Path coefficients	t-value
H _1_	PU -> BI	0.397 ***	7.862
H _2_	PEU -> BI	0.267 ***	4.762
H _3_	SN -> BI	0.180 ***	3.629
H _4_	PREV -> PU	0.499 ***	9.778
H _5_	SE -> PU	0.315 ***	6.468
H _6_	TECH -> PEU	0.400 ***	7.837
H _7_	INN -> PEU	0.334 ***	6.666

*** = p-value 0.001.

The inner model results suggest a significant influence of preventive healthcare awareness and self-efficacy on PU. Further, it shows a significant effect of innovativeness and technology promptness on PEU. The R ^2^ value of PU is 0.534, and PEU is 0.433, showing a moderate predictive relevance of the model. In addition, the outer model results confirm that the PU, PEU, and SN are predictors of intention to adopt technological applications to promote health and prevent diseases.

### 3.4 Importance-performance map analysis (IPMA)

**Figure 3.**
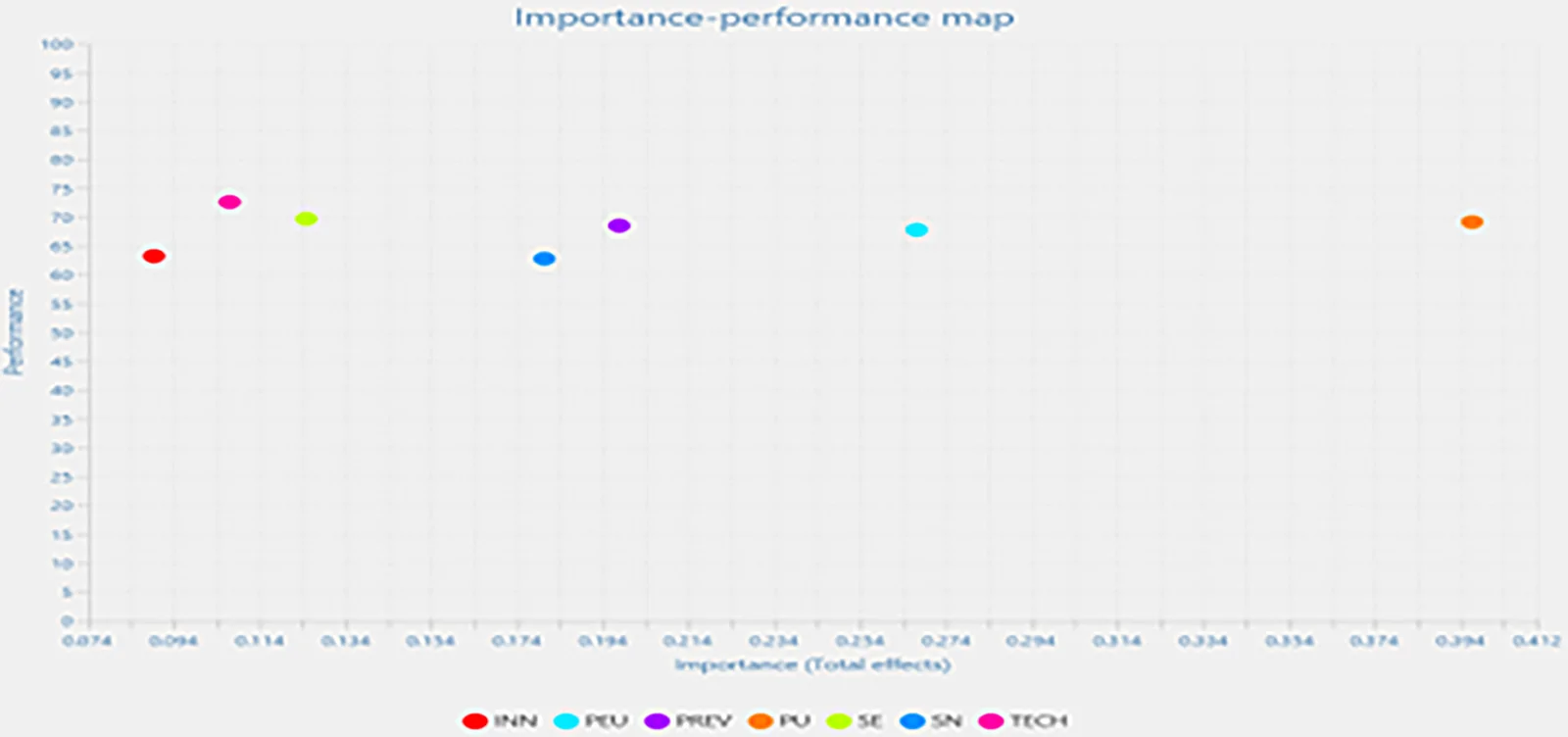
IPMA at construct level.

IPMA is performed to extend the results of PLS-SEM further. It helps to draw conclusions based on the performance and importance of each construct. Thus, it facilitates drawing inferences on two dimensions, importance and performance, to provide administrative implications ( Hair et al., 2017). Hence, IPMA is done to help the target construct BI adopt smart technologies at both construct and indicator levels. The x-axis displays the total effects of the independent variables on the target construct. The y-axis displays the independents’ average construct scores or performance ( Hair et al., 2014). The construct level IPMA results are shown in Figure 3.

**Figure 4.**
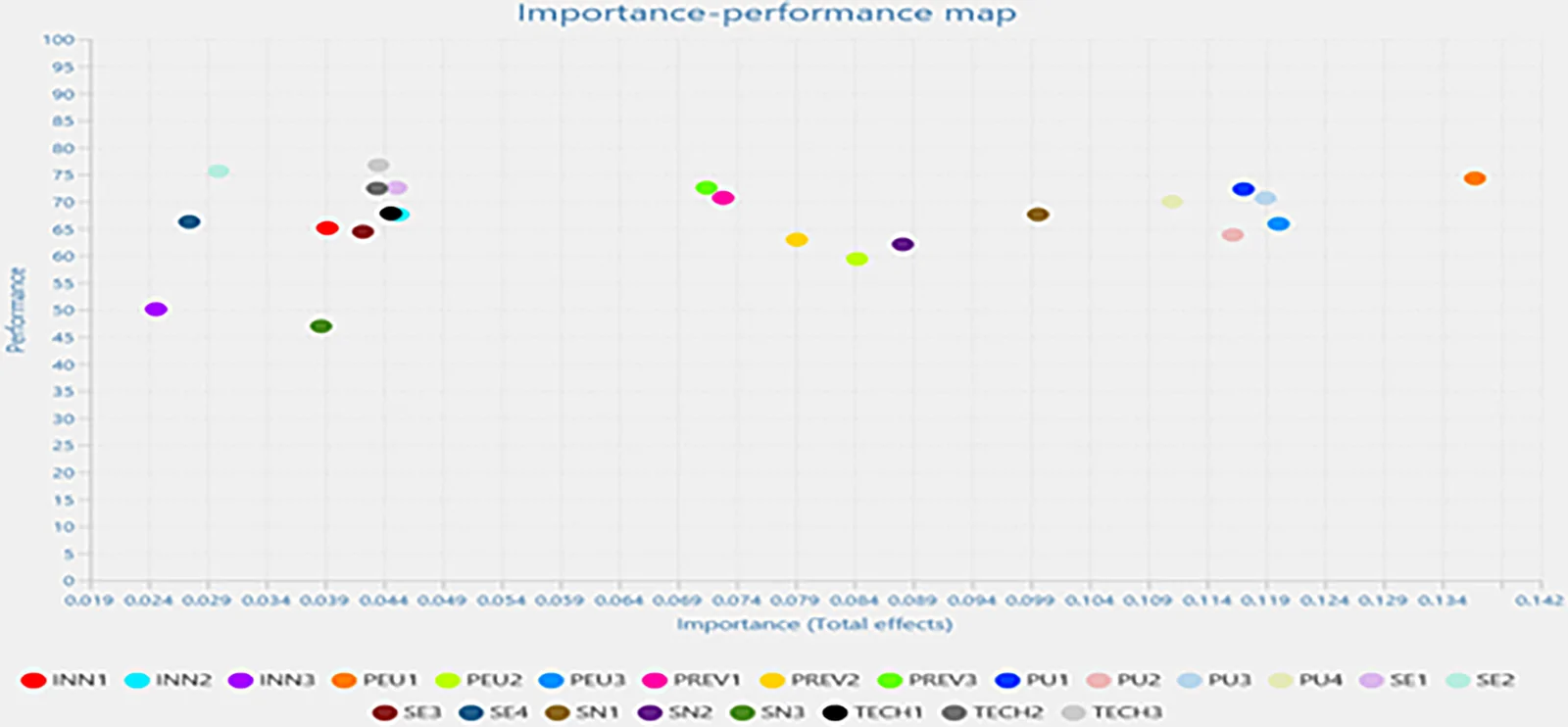
IPMA at the indicator level.

The average performance score (PS) of all the constructs is 67.675, and the average total effect is 0.195. Here, the construct PEU’s performance score (PS) is 67.777, which is slightly greater than the average PS. However, the total effect of PEU on BI is 0.267, which is greater than the average importance score of 0.195. Hence, the PEU construct requires managerial attention to improve the BI to adopt smart technologies. In addition, the construct PU also needs greater attention in enhancing BI as its total effect is 0.395, though the PS is above the average PS of all the constructs. Further, indicator level IPMA is performed to provide more specific insights to improve BI, and the results are shown in Figure 4.

The average PS is 66.878, and the average total effect is 0.071. Hence, it revealed six indicators that went to the prioritization quadrant. They are PEUI “Learning to use technological applications is easy” (β=0.137>0.071, PS=74.295>66.878), PREV1“Awareness on the importance of preventive healthcare services” (β=0.073>0.071, PS=70.705>66.878), PU1 “Using a smart healthcare technology will help in monitor one’s health condition periodically” (β=0.117>0.071, PS=72.308>66.878), PU3 “Using a smart healthcare technology will help to better perform in controlling health issues” (β=0.119>0.071, PS=70.641>66.878), PU4 “I believe that smart healthcare technology would lead to better health outcomes for me” (β=0.111>0.071, PS=70.000 > 66.878), and SN1 “People who are important to me to consider using smart healthcare technology” (β=0.10>0.071, PS=67.628 > 66.878). Hence, these attributes require more attention to improve individuals’ intention to adopt technological applications for health promotion and disease prevention. Further, policymakers and providers of smart healthcare technologies need to be notified of this fact to create awareness among people and enable them to adopt smart healthcare technologies.

## 4. Discussion and Implications

The findings of this study reveal insights into the factors influencing individuals’ behavioural intentions to use technological applications for preventive and promotive healthcare. Our results support the underlying antecedent and determinant factors of the suggested model for adopting smart healthcare technologies among healthcare consumers. This study contributes mainly in three ways: first, it assessed the antecedents of PU of the technological applications; second, it assessed the antecedents of PEU of technological applications; and third, it assessed the determinants of behavioural intentions to adopt technological applications for healthcare.

Our study revealed a positive effect of preventive healthcare awareness and self-efficacy on the PU of technology. This aligns with the previous research findings, which stated preventive healthcare awareness ( Bettiga et al., 2020; Palos-Sanchez et al., 2021) and self-efficacy ( Jokisch et al., 2022) as the key drivers of PU. People who are aware of the importance of disease prevention and who believe in the self-capacity to use technology consider that technology enables services to be more useful.

The study findings reveal a significant effect of technology promptness and innovativeness on perceived ease of use, which confirms them as the antecedents of PEOU, similar to the findings of previous research ( Bettiga et al., 2020; Cheung et al., 2021; Chiu & Cho, 2021). Individuals who are fascinated towards technologies and innovation feel comfortable in the use of smart technologies due to the expertise they have acquired. Hence, healthcare providers can offer enough support to consumers who have less inclination towards new technologies.

Empirical evidence shows a direct effect of perceived usefulness, perceived ease of use and subjective norms on intention to adopt smart technology. Inconsistent with the previous research, perceived usefulness emerges as a significant determinant of individuals’ intentions to adopt smart technologies for healthcare ( Cheung et al., 2021; Chiu & Cho, 2021). In addition, PEOU and SN also have a significant effect on the intention to adopt smart technologies, which is in line with the existing research findings ( Bettiga et al., 2020; Chiu & Cho, 2021). These findings have significant implications for policymakers and providers, indicating that enhancing perceived ease of use and social desirability, in addition to perceived usefulness, is essential to increasing adoption intentions of technological applications. Even though providing a user-friendly technology and interface are critical determinants of the intention to accept smart health technologies, it is insufficient to encourage widespread adoption. Hence, technology providers should go beyond merely reducing system complexity; they must actively communicate and promote the utility of preventive healthcare systems to both the target individuals and their social networks. This approach ensures that the benefits of technology are understood and valued within the broader community, enhancing overall acceptance and use.

These findings will be useful for healthcare providers, policymakers, researchers, and health technology marketers who are diffusing technological applications to promote health and prevent diseases. It can guide healthcare providers in implementing and promoting these technologies within their practice. Implementing user-friendly technological solutions based on study findings can improve patient engagement and healthcare delivery. Further, policymakers can use the study findings to inform the development of policies and regulations that support the adoption and integration of technological applications for health promotion and disease prevention. The study findings will be helpful for health technology marketers to tailor marketing strategies and messages that emphasize the benefits and usability of their products for preventive and promotive healthcare. Moreover, this study may benefit researchers who want to build upon the findings to conduct further investigations into specific factors influencing technology adoption in healthcare.

## 5. Conclusion and future research recommendations

This research offers insights into the antecedent and determinant factors driving the adoption of technologies in healthcare. The study provides a foundation for future research on the effectiveness and sustainability of technological interventions in preventive and promotive healthcare. Overall, the study’s implications highlight the importance of collaboration among stakeholders to promote the adoption and diffusion of technological applications for health promotion and disease prevention. By translating research findings into actionable strategies, stakeholders can collectively contribute to improving population health outcomes and advancing healthcare delivery through innovative technologies. Despite the valuable findings, the study has limitations that suggest future research in this domain. First, there is a limitation regarding the generalizability of the findings, as this study used convenience sampling, a non-probability sampling method. Hence, future researchers are recommended to perform the research using the probability sampling method. Second, this study has taken an opinion on the acceptance of overall general smart healthcare technologies such as mhealth/mobile health, telehealth, online medicine purchases, and wearables like smartwatches etc. Future studies could be done on the specific smart healthcare technology to gain insights into respective technological applications. Third, this study has used a quantitative research approach, which often assumes objectivity, potentially overlooking the subjective nature of data collection and analysis processes. Hence, future research can be done using mixed methods, such that the qualitative approach may identify concerns people have if any, other than those that the study constructs. Further, as this study is performed in the Indian context, such studies can be performed in other countries’ contexts to gain more insights into adoption intention, as people might have different knowledge, opinions, and perceptions depending on the cultural and socioeconomic exposures and accessibility to smart technology.

## Ethics and consent

The complete protocol was submitted to the Institutional Ethics Committee-2 of Kasturba Medical College and Kasturba Hospital and received the approval on September 8, 2023 with the number IEC2:448/2023. Further, the study was registered with the Clinical Trial Registry of India, which subsequently approved the study’s conduct. After receiving approval, the questionnaire was sent to people of all ages using open media platforms, including email, LinkedIn, and WhatsApp. This study adhered to the ethical principles outlined in the Declaration of Helsinki. Participation in the survey was entirely voluntary, and informed written consent was obtained from all respondents prior to their participation.

## Author contributions

• **Debanjali Jairam:** Conceptualization, Data curation, Formal analysis, Investigation, Methodology, Project administration, Writing – original draft
• **Dr Swathi K S:** Conceptualization, Data curation, Formal analysis, Investigation, Methodology, Supervision, Writing – original draft
• **Dr Smitha Nayak:** Conceptualization, Formal analysis, Writing – review and editing

## Data Availability

Figshare: Perception of smart technology adoption data.xlsx, https://doi.org/10.6084/m9.figshare.28016792.v1 ( Jairam et al., 2024). This project contains the following underlying data:
• Perception of smart technology adoption dataset.xlsx Data are available under the terms of the Creative Commons Attribution 4.0 International license (CC-BY 4.0). Figshare: Study tool – Smart Technology adoption ( Jairam et al., 2025). This project contains the following underlying data:
• Questionnaire – Smart Technology Adoption Intention.pdf Data are available under the terms of the Creative Commons Attribution 4.0 International license (CC-BY 4.0).

## Software availability statement

Smart PLS 4 is a subscription-based software; however, one can use R statistical software, which is a free software for PLS SEM analysis.
