# Supplementary material for: Embracing smart wellness: Exploring perceptions of preventive healthcare technology in the digital era
Source: F1000Res. 2025 Sep 2;14:125. Originally published 2025 Jan 24. [Version 3] doi: 10.12688/f1000research.160126.3 (PMC13555066; doi:10.12688/f1000research.160126.3)
Supplement: Peer review history

# Reviewer report: Reviewer response for version 3

**DOI:** 10.5256/f1000research.187232.r497866

**Published:** 2026-08-06

**Authors:** Neha Sadhotra

**Affiliations:** 1 Monjee Institute of Management Studies (NMIMS) Deemed-to-be-University, Mumbai, Maharashtra, India

This cross-sectional study examines the antecedents of behavioural intention to adopt smart healthcare technologies (wearables, mHealth, telemedicine, e-health) for preventive and promotive health in the Indian context. Drawing on the Technology Acceptance Model, extended with constructs from the Health Belief Model, the authors test a model in which Prevention Awareness and Self-Efficacy act as antecedents of Perceived Usefulness; Technology Promptness and Innovativeness act as antecedents of Perceived Ease of Use; and Perceived Usefulness, Perceived Ease of Use, and Subjective Norms directly predict Behavioural Intention. Data from 390 respondents were analysed using PLS-SEM in SmartPLS 4, supplemented by an Importance-Performance Map Analysis. All seven hypotheses were supported. The measurement model met standard reliability and validity thresholds, and the R² for Behavioural Intention was 0.492. The authors conclude that self-efficacy, preventive awareness, technology promptness, innovativeness, and social influence play significant roles in adoption, and they draw implications for providers, policymakers, developers, and marketers.

*Points that must be addressed to make the article scientifically sound*

1. **Overstated causal language relative to the design (conclusions- Partly).** The manuscript uses causal verbs throughout ("influence," "drive," "impact") that a cross-sectional, single-instrument, self-report design cannot support. Please either soften the language to associational terms or explicitly frame the causal ordering as theory-derived and untested. This is the single most important revision for scientific soundness.
2. **Common method variance is not assessed (statistical analysis- Partly).** All constructs, predictors and outcome alike, were measured with one self-report questionnaire at one time point, which raises a real risk of common method bias. A Harman's single-factor test, a full collinearity assessment (inner-model VIFs), or a marker-variable approach is inexpensive to run and should be reported. This is a required addition given the all-positive result pattern.
3. **Generalizability claims exceed what the sample supports (presentation- Partly).** The paper describes the population as "the general public in India," but the realised sample is roughly 66% aged 36–50 and ~98% graduate/postgraduate, recruited by convenience. The authors acknowledge the >50 gap (only ~8%), but the education skew is not addressed at all and matters greatly for an adoption study, since lower-education and older groups are where barriers concentrate. Please add the education-skew limitation explicitly and temper generalisation claims so conclusions are scoped to educated, middle-aged, digitally engaged respondents.

*Points that would strengthen the article*

1. **Novelty framing.** The contribution is largely geographic (a TAM/HBM replication in the Indian context) rather than theoretical. Seven uniformly positive hypotheses, with no moderators, competing predictions, or null expectations, limit the theoretical yield, a concern one reviewer already raised. The paper would be stronger if it stated its contribution modestly and precisely rather than implying a broader advance.
2. **Sub-threshold reliability handled transparently.** Two constructs, Perceived Ease of Use (α = 0.659) and Subjective Norms (α = 0.657), fall below the conventional 0.70 Cronbach's alpha, yet the text states all alphas exceed 0.7. Please correct this and justify retention explicitly on the basis of composite reliability and AVE, rather than glossing over it.
3. **Unexplained variance.** With R² for Behavioural Intention at 0.492, over half the variance is unexplained. The "predictive relevance" framing should be balanced by acknowledging the constructs omitted (e.g., trust, privacy concern, cost, facilitating conditions) that the adoption literature consistently finds relevant.
4. **Literature currency and accuracy (why "Partly" on presentation).** Two of three reviewers flagged citation of current literature as insufficient. In addition, a residual grammatical error the reviewer identified ("In order to close this gap, this study looks at hence, this study enables us to better…") is still present in the version 3 introduction and should be fixed. A careful proof pass is needed.
5. **IPMA is a genuine strength** and could be foregrounded more. The importance-performance findings (that PU and PEU, and specific indicators such as ease of learning and periodic health monitoring, warrant managerial priority) are the most actionable part of the paper and differentiate it from a standard TAM replication.

*Assessment of statistical analysis*

 The PLS-SEM workflow is appropriate and correctly executed for what it does: reflective measurement model assessment (α, CR, AVE), Fornell-Larcker discriminant validity, 5000-sample bootstrap, and reported path coefficients with t-values all above 1.96 at p < 0.001. The reason for "Partly" rather than "Yes" is not the mechanics but the interpretation: the absence of a common-method-variance check, the missing HTMT ratio (now generally preferred over Fornell-Larcker for discriminant validity), and the causal reading of associational estimates. Addressing points 1 and 2 above would move this to "Yes."

*Reproducibility*

 The underlying dataset and the study instrument are openly deposited on Figshare under CC-BY, and the software and analytical steps are described in enough detail to allow replication, so source-data availability and methods detail are satisfactory.

*Overall*

 This is a competently executed, honestly reported, and openly documented adoption study with a useful IPMA component and a meaningful regional descriptive contribution. Its limitations are those of a convenience-sampled, cross-sectional, self-report TAM design, and the main required revisions are to align the causal language with the design, test for common method bias, and scope the generalizability claims to the actual sample.

Is the work clearly and accurately presented and does it cite the current literature?

Partly

If applicable, is the statistical analysis and its interpretation appropriate?

Partly

Are all the source data underlying the results available to ensure full reproducibility?

Yes

Is the study design appropriate and is the work technically sound?

Yes

Are the conclusions drawn adequately supported by the results?

Partly

Are sufficient details of methods and analysis provided to allow replication by others?

Yes

Reviewer Expertise:

Technology adoption in healthcare

I confirm that I have read this submission and believe that I have an appropriate level of expertise to confirm that it is of an acceptable scientific standard, however I have significant reservations, as outlined above.

# Reviewer report: Reviewer response for version 3

**DOI:** 10.5256/f1000research.187232.r497865

**Published:** 2026-07-16

**Authors:** S.K. NAGARAJAN NAGARAJAN

**Affiliations:** 1 Annamalai University, Chidambaram, Tamil Nadu, India

Author(s) had done a good work and need of the hour. 

**Introduction **: As the study was restricted to Indian context, few healthcare, medical data on Indian context will add more value to the reader and focused.

 Page 4, Para 2 : Line 2 : In order to close this gap, this study looks at hence, this study enables us to better (Need reframing) 

 In Table-1, Occupation - Professional - is confusing among the choices given.

 Page 9 : Table - 4 : Usually table is given below the exact reference location for easy ready reference.  Here it was tabulated early.  To make to reader friendly and also there is possibility of giving in exact location think of realigning.

**Conclusion **: Rather than providing general conclusive remarks, if extracted from the research done and fruitful directions for policy makers, industry experts, users, healthcare professionals it will be more meaningful and value addition.

Is the work clearly and accurately presented and does it cite the current literature?

No

If applicable, is the statistical analysis and its interpretation appropriate?

I cannot comment. A qualified statistician is required.

Are all the source data underlying the results available to ensure full reproducibility?

Yes

Is the study design appropriate and is the work technically sound?

Yes

Are the conclusions drawn adequately supported by the results?

Partly

Are sufficient details of methods and analysis provided to allow replication by others?

Yes

Reviewer Expertise:

Management, Software Product Marketing

I confirm that I have read this submission and believe that I have an appropriate level of expertise to confirm that it is of an acceptable scientific standard.

# Reviewer report: Reviewer response for version 3

**DOI:** 10.5256/f1000research.187232.r411196

**Published:** 2025-09-17

**Authors:** Catharina M van Leersum

**Affiliations:** 1 University of Twente, Enschede, Netherlands Antilles; 2 Open University of The Netherlands (Ringgold ID: 10198), Heerlen, Limburg, The Netherlands

I see how the authors have used the comments to revise their manuscript. I have no further comments to make.

Is the work clearly and accurately presented and does it cite the current literature?

Partly

If applicable, is the statistical analysis and its interpretation appropriate?

Yes

Are all the source data underlying the results available to ensure full reproducibility?

Yes

Is the study design appropriate and is the work technically sound?

Yes

Are the conclusions drawn adequately supported by the results?

Yes

Are sufficient details of methods and analysis provided to allow replication by others?

Yes

Reviewer Expertise:

AI, eHealth, STS

I confirm that I have read this submission and believe that I have an appropriate level of expertise to confirm that it is of an acceptable scientific standard.

# Reviewer report: Reviewer response for version 2

**DOI:** 10.5256/f1000research.180643.r399445

**Published:** 2025-08-19

**Authors:** Catharina M van Leersum

**Affiliations:** 1 University of Twente, Enschede, Netherlands Antilles; 2 Open University of The Netherlands (Ringgold ID: 10198), Heerlen, Limburg, The Netherlands

Dear authors, your manuscript discusses a relevant topic and is based on a well conducted and documented method. To make the paper more attractive to readers, I suggest some comments for improvement of presenting the study in your manuscript, especially in the first part. Hopefully these advices can assist you in preparing an adjusted manuscript.

 Introduction

1. The introduction is very informative, but it does not really inform me as a reader why I should read this paper and what you as a researcher are aiming to do. Make clear why I should read this paper and what is relevant about all the details/information provided. The last paragraph is not really informative since it does not provide any information which could not be expected by the reader. This could be removed.
2. Minor remark: you use quite some capitals where these are not needed.
3. Minor remark: please provide the full writing of IoT before providing/using the acronym.

Literature and hypotheses

1. I am not sure about the presentation of the different hypotheses. These are all going to discuss the ‘positive impact’ and that is fine, but then it would not be necessary in my opinion to make this into seven different hypotheses. Please diminish this use of words and use them differently. Because you mention the bulk of literature, but do not really discuss this and then suddenly present figure 1. I would like to get more information on figure 1 instead of all information you spread regarding seven not very interesting hypotheses. Start this section with this figure and then show how it informs your research and how you reach to the hypothesis that all the elements of TAM and HBM (or name the seven items specifically) will have a positive impact.
2. Minor remark: you gave some acronyms like PU and PEOU, but then still use the full writing in some later occurrences. This is not needed and could be removed.

Results

1. I notice that there is a lack of information about the 50 years and older population. Could you please reflect on the lack of information about this older population. This might be valuable since this population uses healthcare quite often.
2. Figure 2 is not really informative and only leave it with table 4 would be fine. However, it would be good to rename H1-7 and only leave it with the relations. Especially if you take my earlier comment into account.
3. Minor remark: again with the acronyms. Here you use more acronyms for the seven items. Please start using the acronyms from the first instance you mention them. This is in the literature and hypotheses part, where you now only introduced PU and PEOU, but you already mention the wordings of all other acronyms. Be consistent is my message.
4. Minor remark or question: PEU is the same as PEOU?

Discussion

1. From the start of the discussion I see the aim of this research. Thank you, and it would be great to read something about this earlier, see my first comment.
2. It would be great to read a bit about your considerations towards the strengths and especially some limitations. See also my earlier comment regarding lack of data from the older population.

Conclusions

1. In this part as well as in the discussion part you talk a bit about future considerations and future research. Please combine these sections. I would prefer to read this at the end of the discussion, where you now already indeed mention something about this. Thus remove the future research from the conclusion back to the discussion and possibly this can assist you in overcoming to mention things twice.

Is the work clearly and accurately presented and does it cite the current literature?

Partly

If applicable, is the statistical analysis and its interpretation appropriate?

Yes

Are all the source data underlying the results available to ensure full reproducibility?

Yes

Is the study design appropriate and is the work technically sound?

Yes

Are the conclusions drawn adequately supported by the results?

Yes

Are sufficient details of methods and analysis provided to allow replication by others?

Yes

Reviewer Expertise:

healthcare, artificial intelligence, eHealth, older adults

I confirm that I have read this submission and believe that I have an appropriate level of expertise to confirm that it is of an acceptable scientific standard, however I have significant reservations, as outlined above.

# Response

**Published:** 2025-08-25

**Authors:** Swathi K S

**Affiliations:** Prasanna School of Public Heal, Manipal Academy of Higher Education, Manipal, Karnataka, India

Dear Reviewer, 

 Thank you for the valuable suggestions for improving the quality of this manuscript. The responses to the comments are given below.

 Introduction

1. The introduction is very informative, but it does not really inform me as a reader why I should read this paper and what you as a researcher are aiming to do. Make clear why I should read this paper and what is relevant about all the details/information provided. The last paragraph is not really informative since it does not provide any information which could not be expected by the reader. This could be removed.

Minor remark: you use quite some capitals where these are not needed.

**Response: **Thank you for the comment. The value added by the research has been added to the introduction section of the manuscript. This clearly explains “why” a researcher should read our article.

 In order to close this gap, this study looks at Hence, this study enables us to better understand the preferences and expectations, enabling developers to enhance user experience and engagement with smart technologies. It also helps us to identify psychological, social, and technical barriers that impede the widespread adoption of smart health technologies. This is explored in the backdrop of the Technology Acceptance Model (TAM). ). The Technology Acceptance Model (TAM) provides a theoretical foundation for the research endeavour and enables researchers to explore the interplay of two important antecedents of technology adoption (perceived usefulness and perceived ease of use) in the context of smart wellness products.

**2. Minor remark: please provide the full writing of IoT before providing/using the acronym.**

**Response: **Noted and necessary changes have been incorporated. 

 Reviewer comments : Literature and hypotheses

1. I am not sure about the presentation of the different hypotheses. These are all going to discuss the ‘positive impact’ and that is fine, but then it would not be necessary in my opinion to make this into seven different hypotheses. Please diminish this use of words and use them differently. Because you mention the bulk of literature, but do not really discuss this and then suddenly present figure 1. I would like to get more information on figure 1 instead of all information you spread regarding seven not very interesting hypotheses. Start this section with this figure and then show how it informs your research and how you reach to the hypothesis that all the elements of TAM and HBM (or name the seven items specifically) will have a positive impact

**Response:** Thank you for the review. We have revisited the hypothesis statements and incorporated the changes wherever necessary.

        2. Minor remark: you gave some acronyms like PU and PEOU, but then still use the full writing in some later occurrences. This is not needed and could be removed. 

**Response: **We have included the necessary changes in the manuscript.

**Results**

1. **I notice that there is a lack of information about the 50 years and older population. Could you please reflect on the lack of information about this older population? This might be valuable since this population uses healthcare quite often.**

**Response: **Yes we agree that the population above 50 displays an inclination to use smart technologies and we have only 8% of our responses in the cluster. When this study was conceptualized, “age” was not considered as a moderator and hence a representative sample was not drawn from each cluster. However, we have mentioned this in our limitations as a direction for future researchers to explore.

**2. Figure 2 is not really informative and only leave it with table 4 would be fine. However, it would be good to rename H1-7 and only leave it with the relations. Especially if you take my earlier comment into account.**

**Response:** Figure 2 is the structural model adopted in this study and hence, researchers have decided to retain both figure 2 and table 4.

**3. Minor remark: again with the acronyms. Here you use more acronyms for the seven items. Please start using the acronyms from the first instance you mention them. This is in the literature and hypotheses part, where you now only introduced PU and PEOU, but you already mention the wordings of all other acronyms. Be consistent is my message.**

**Response:** Thank you for the observation. Necessary changes have been incorporated.

       4. Minor remark or question: PEU is the same as PEOU?

**Response: **Yes. We apologize for the typo error. We have made the necessary changes in the manuscript.  

**Discussion**

1. **From the start of the discussion I see the aim of this research. Thank you, and it would be great to read something about this earlier, see my first comment.**

**Response:** We have incorporated the same in the introduction section.

**2. It would be great to read a bit about your considerations towards the strengths and especially some limitations. See also my earlier comment regarding lack of data from the older population.**

**Response:** We have mentioned this is the limitations section of the manuscript.

**Conclusions**

**In this part as well as in the discussion part you talk a bit about future considerations and future research. Please combine these sections. I would prefer to read this at the end of the discussion, where you now already indeed mention something about this. Thus remove the future research from the conclusion back to the discussion and possibly this can assist you in overcoming to mention things twice.**

**Response: **Thank you for the suggestion. We have aligned the manuscript according to established norms and journal considerations. Hence, the conclusion section not only presents the final comments but clearly spells out direction for future researchers. This has been placed in the last section of the manuscript.

# Reviewer report: Reviewer response for version 1

**DOI:** 10.5256/f1000research.175959.r373523

**Published:** 2025-04-01

**Authors:** Santosh Kumar

**Affiliations:** 1 Koneru Lakshmaiah Education Foundation, Vaddeswaram, India

Some of the suggestions that could improve the quality of this work include:

1. Sampling from convenient sites may introduce bias To enhance generalizability, exploratory studies can also adopt probability sampling techniques and larger representative, population-based studies should further investigate these findings.
2. Discuss the few WaveFlex Biosensors in the literature survey to get interest in the readers. 
3. Broaden the research to include culture comparisons; as well as comparing perceptions in regions with different health care systems and different access to technologies.
4. Instead of conducting a holistic review, study smart healthcare technologies like wearables, tele health, or mHealth apps.
5. Pair it with qualitative methods — interviews or focus groups — that can help flesh out user issues and attitudes beyond the numbers.
6. Develop a longitudinal research method to see how technology adoption behavior changes over time, including with an external force snipe like a policy or technology change.
7. To ascertain whether adopting smart health technologies translates into health benefits, include objective health outcomes.
8. Formulate actionable recommendations for practitioners and/or technology designers on how to better reach and engage end-users.
9. More consideration of possible biases due to use of self-reported data and triangulation of findings with either objective health data or application usage metrics
10. Develop a technology readiness index (TRI) based on the extent to which respondents correspond to readiness for smart healthcare technology use and capability to adapt to smart healthcare technology.

Is the work clearly and accurately presented and does it cite the current literature?

No

If applicable, is the statistical analysis and its interpretation appropriate?

Partly

Are all the source data underlying the results available to ensure full reproducibility?

Partly

Is the study design appropriate and is the work technically sound?

Partly

Are the conclusions drawn adequately supported by the results?

No

Are sufficient details of methods and analysis provided to allow replication by others?

Partly

Reviewer Expertise:

Sensor

I confirm that I have read this submission and believe that I have an appropriate level of expertise to confirm that it is of an acceptable scientific standard, however I have significant reservations, as outlined above.

# Response

**Published:** 2025-04-14

**Authors:** Swathi K S

**Affiliations:** Prasanna School of Public Heal, Manipal Academy of Higher Education, Manipal, Karnataka, India

Response to Reviewer’s Comments

 1. Sampling from convenient sites may introduce bias To enhance generalizability, exploratory studies can also adopt probability sampling techniques and larger representative, population-based studies should further investigate these findings.

 Response:

 We acknowledge your valuable observation regarding the potential bias introduced by convenience sampling. As this study was exploratory in nature, convenience sampling was adopted to gain initial insights within the constraints of available resources and accessibility. We agree that this may limit the generalizability of the findings. Hence, we have mentioned it as one of the limitations of this study in the conclusion section and included a recommendation for future research using a more representative sampling method.

 2. Discuss the few WaveFlex Biosensors in the literature survey to get interest in the readers. 

 Response:

 In response to your suggestion, we have added the literature on WaveFlex biosensors that have been reported in recent studies in the second paragraph of introduction section while discussing about the recent advancements. This addition highlights the practical relevance and innovation in wearable smart technology.

 3. Broaden the research to include culture comparisons; as well as comparing perceptions in regions with different health care systems and different access to technologies.

 Response:

 Thank you for this insightful suggestion. We acknowledge the importance of incorporating cultural comparisons and examining regional differences in healthcare systems and access to technology. In the revised manuscript, we have expanded the discussion to include a cross-cultural perspective on the adoption of smart technology for preventive healthcare.

 4. Instead of conducting a holistic review, study smart healthcare technologies like wearables, telehealth, or mHealth apps.

 Response:

 We appreciate the reviewer’s suggestion to focus specifically on smart healthcare technologies such as wearables, telehealth, or mHealth apps. Our intention was to capture the broader landscape of smart technologies in preventive healthcare and understand their interrelated roles in fostering proactive health management. However, in future research, we will focus on a specific technological application such as wearables, telehealth or mHealth, and we have mentioned it in the future research scope section in the concluding paragraph.

 5. Pair it with qualitative methods — interviews or focus groups — that can help flesh out user issues and attitudes beyond the numbers.

 Response:

 Thank you for this valuable suggestion. We appreciate the insight that integrating qualitative methods such as interviews or focus groups can provide a more nuanced understanding of user issues and attitudes toward smart technology in preventive healthcare. While the current study focused on a quantitative empirical approach to examine broad patterns and associations, we acknowledge the importance of complementing this with qualitative insights. Hence, we have added a note in the limitations and future research scope section, highlighting the potential for future studies to adopt a mixed-methods approach.

 6. Develop a longitudinal research method to see how technology adoption behavior changes over time, including with an external force snipe like a policy or technology change.

 Response:

 Yes this is a perfect research gap that we have already initiated work. A longitudinal research design would enable us to compare adoption, which is our next phase of research. However, we have adopted this manuscript a cross-sectional approach that enabled us to capture the intention to adopt small wellness, laying a foundation for our subsequent project.

 7. To ascertain whether adopting smart health technologies translates into health benefits, include objective health outcomes.

 Response:

 This is a valid suggestion, and we thank you for it. Health outcomes can be very broad, and we need to narrow down on which health outcomes we are focusing on ( fitness, weight, sugar levels, wellness etc.). As this is a behavioural study, we have not included it in the scope of the study. Further studies can work on this gap, and we have incorporated it in the direction for the future research section of the manuscript.

 8. Formulate actionable recommendations for practitioners and/or technology designers on how to better reach and engage end-users.

 Response:

 Thank you for the observation. We have incorporated our suggestions in the implications paragraph of the discussion and implications section in the revised manuscript.

 9. More consideration of possible biases due to use of self-reported data and triangulation of findings with either objective health data or application usage metrics.

 Response:

 As mentioned in our response to review comment no 2, the objective of the manuscript to capture the behavioural dimension and not impact on clinical outcome. In social science research, established Likert scales are adopted to capture intention, and hence, we proceeded with this study after validating the questionnaire with a pilot study. We have incorporated the same in the last paragraph of the Method section of the manuscript.

 10. Develop a technology readiness index (TRI) based on the extent to which respondents correspond to readiness for smart healthcare technology use and capability to adapt to smart healthcare technology.

 Response:

 The TRI  is a tool for measuring technology readiness by evaluating users' acceptance and capability in using new technologies across four core dimensions (optimism, innovativeness, discomfort, and insecurity) as proposed by Parasuraman(2000). All four dimensions have not been incorporated in this manuscript as this research was conceived to measure intention to embrace smart wellness The research team unanimously opined that the respondent segment we were targeting was aware and into the adoption of the technology innovation adoption cycle. However, future researchers can explore this, and hence, it has been incorporated as a direction for future research.

 Thank you
